# Serum Biomarker Panel for Diagnosis and Prognosis of Pancreatic Ductal Adenocarcinomas

**DOI:** 10.3389/fonc.2021.708963

**Published:** 2021-07-05

**Authors:** Shreya Mehta, Nazim Bhimani, Anthony J. Gill, Jaswinder S. Samra, Sumit Sahni, Anubhav Mittal

**Affiliations:** ^1^ Northern Clinical School, Faculty of Medicine and Health, University of Sydney, Sydney, NSW, Australia; ^2^ Kolling Institute of Medical Research, University of Sydney, Sydney, NSW, Australia; ^3^ Upper Gastro Intestinal (GI) Surgical Unit, Royal North Shore Hospital and North Shore Private Hospital, Sydney, NSW, Australia; ^4^ Cancer Diagnosis and Pathology Group, Kolling Institute of Medical Research, Royal North Shore Hospital, St Leonards, NSW, Australia; ^5^ Australian Pancreatic Centre, Sydney, NSW, Australia; ^6^ NSW Health Pathology, Department of Anatomical Pathology, Royal North Shore Hospital, St Leonards, NSW, Australia

**Keywords:** pancreatic ductal adenocarcinoma, diagnostic biomarkers, prognostic biomarkers, S100A4, Ca-125 and Ca 19-9, survival analysis

## Abstract

**Background:**

Patients with pancreatic ductal adenocarcinoma (PDAC) have late diagnosis which results in poor prognosis. Currently, surgical resection is the only option for curative intent. Identifying high-risk features for patients with aggressive PDAC is essential for accurate diagnosis, prognostication, and personalised care due to the disease burden and risk of recurrence despite surgical resection. A panel of three biomarkers identified in tumour tissue (S100A4, Ca125 and Mesothelin) have shown an association with poor prognosis and overall survival. The diagnostic and prognostic value of the serum concentration of this particular biomarker panel for patients with PDAC has not been previously studied.

**Methods:**

Retrospectively collected blood samples of PDAC patients (n =120) and healthy controls (n =80) were evaluated for the serum concentration of select biomarkers – S100A4, S100A2, Ca-125, Ca 19-9 and mesothelin. Statistical analyses were performed for diagnostic and prognostic correlation.

**Results:**

A panel of four biomarkers (S100A2, S100A4, Ca-125 and Ca 19-9) achieved high diagnostic potential (AUROC 0.913). Three biomarkers (S100A4, Ca-125 and Ca 19-9) correlated with poor overall survival in a univariable model (*p < 0.05*). PDAC patients with abnormal levels of 2 or more biomarkers in their serum demonstrated significantly lower survival compared to patients with abnormal levels of one or less biomarker (*p < 0.05*).

**Conclusion and Impact:**

The identified biomarker panels have shown the potential to diagnose PDAC patients and stratify patients based on their prognostic outcomes. If independently validated, this may lead to the development of a diagnostic and prognosticating blood test for PDAC.

## Introduction

Pancreatic ductal adenocarcinoma (PDAC) has the highest mortality of all major cancers and is projected to become the second most common cause of cancer related death by 2030 (1, 2). Currently, clinical decision making is based on the radiological staging, vascular involvement, overall disease burden and the patient’s premorbid status. However, current algorithms used to treat PDAC do not specifically take into account the biological behaviour of each tumour. Recent work on the genetic variability and biology of PDAC highlights the importance of tumour biology in chemosensitivity and overall survival (3, 4). Hence, there is an urgent need for an easily quantifiable and cost-effective biomarker signature to assist clinicians in taking informed treatment decisions based on an individual patient’s tumor biology.

A PDAC tissue biomarker panel (S100A4, Mesothelin, Ca-125) approach has recently been shown to be successful in prognosticating pancreatic cancer outcome (5). The expression of these biomarkers in the tumour tissue has been shown to track with the genetic changes associated with a more aggressive (so called ‘squamous’) genotype of PDAC (6). However, given that these tissue sample may be difficult to obtain at first presentation, a liquid biopsy using secreted biomarkers in the patient’s blood would be beneficial in diagnosis and prognostication with personalisation of treatment for patients with PDAC.

The aim of this study was to: (1) determine the diagnostic potential of this set of biomarkers individually and as a panel by comparing serum expression of these biomarkers in PDAC patients and healthy controls; (2) establish a “normal” and “abnormal” result for the expression of a set of biomarkers – Ca 19.9, Ca-125, Mesothelin, S100A2 and S100A4 by comparing serum values in patients with PDAC with healthy controls; (3) identifying the prognostic significance of these biomarkers based on serum expression in PDAC patients and correlation with overall survival.

## Methodology

### Patient Information

Patients across two tertiary centres, Royal North Shore Hospital and North Shore Private hospital, who had surgically resected PDAC, and serum collected at the time of surgery from 2007 – 2014 were included in this study. The serum was obtained from the Kolling Tumour Bank. Serum from age and sex matched healthy controls was also obtained from the Kolling Tumour Bank.

### Standard Protocol Approvals, Registrations, and Patient Consents

Ethical approval was obtained at the respective tertiary centres (references HREC/16/HAWKE/105 and NSPHEC 2016-007). Informed written consent was obtained from all participants and/or their designated surrogate. Northern Sydney Local Health District reference: RESP/16/76.

### ELISA Assay

ELISA assay was performed for S100A4 (Circulex S100A4 ELISA Kit Version 2 Cat# CY-8086, MBL Life Science, Japan), S100A2 (Cat# SEC009Hu, Cloud-clone Corp, Wuhan, China) and Mesothelin (Mesomark ELISA Kit; Fujirebio Diagnostics, PA, USA), following manufacturer’s guidelines. Each sample was performed in duplicate. Assay for Ca-125 and Ca 19-9 was performed at the Pathology North, RNSH, using their standard protocol.

### Data Analysis

The biomarker concentrations between PDAC and Healthy Controls were compared and analysed for diagnostic potential using AUROC curves. The cut-off values used for multivariable analysis (**Supplementary Table 1**) were determined based on optimum sensitivity and specificity in univariable analysis.

Patient characteristics were compared for survival using Cox Proportional Hazard Model. Univariable survival analysis for biomarkers was performed using Kaplan-Meier curves, and statistical significance was achieved using the Log-rank test with a *p*-value <0.05. The biomarker cut-off values for the survival analysis were based on either clinically used values or values where high specificity was achieved with minimum loss of sensitivity (**Supplementary Table 2**). PDAC patients were divided into two groups based on normal or abnormal levels of biomarker concentration. The cut-offs were established based on the observed direction of change in biomarkers. Biomarkers with significantly elevated levels in the serum of PDAC patients compared to healthy controls (*i.e.*, S100A2, Ca-125, Mesothelin and Ca 19-9), patients were classified with abnormal levels when their biomarker level was more than cut-off. In contrast, for the biomarkers with significantly decreased levels in PDAC serum, patients were classified with abnormal levels when their biomarker level was less than cut-off. All statistical analyses were performed using Stata SE, IBM SPSS Statistics for Windows 2019 (Version 26.0 Armonk, NY, IBM Corp) or GraphPad Prism Software (Version 8.4.2).

## Results

### Population Demographics

Patient characteristics are described in **Table 1**. There were 120 patients with PDAC included in this study and 80 healthy controls.

**Table 1:** Patient and tumour characteristics and correlation with survival status.

	Total n (%)	HR	95% CI	*p*-value
Age				0.448
<70 years	73 (60.8)	Reference		
≥70 years	47 (39.2)	1.17	0.78-1.78	
Gender				0.510
Male	61 (50.8)	Reference		
Female	59 (49.2)	0.87	0.58-1.31	
Tumour size				0.006
<35mm	55 (45.8)	Reference		
≥35mm	65 (54.2)	1.80	1.18-2.73	
T Stage				0.221
T1 & T2	9 (7.5)	Reference		
T3 & T4	111 (92.5)	1.68	0.73-3.84	
Node Positive				0.003
No	26 (21.7)	Reference		
Yes	94 (78.3)	2.44	1.35-4.41	
Vascular Invasion				0.002
No	46 (38.3)	Reference		
Yes	74 (61.7)	1.99	1.28-3.08	
Perineural Invasion				0.072
No	38 (31.7)	Reference		
Yes	82 (68.3)	1.52	0.96-2.39	
Grade				0.014
0 or 1	84 (70)	Reference		
2 or 3	36 (30)	1.74	1.12-2.69	
Blood loss				0.140
<450mL	52 (43.3)	Reference		
≥450mL	68 (56.7)	1.37	0.90-2.09	
Length of stay				0.509
<12 days	45 (37.5)	Reference		
≥12 days	75 (62.5)	0.87	0.57-1.32	
Margin Status				0.002
R0	49 (40.8)	Reference		
R1	71 (59.2)	1.97	1.28-3.03	

### Diagnostic Biomarkers

**Figure 1:**
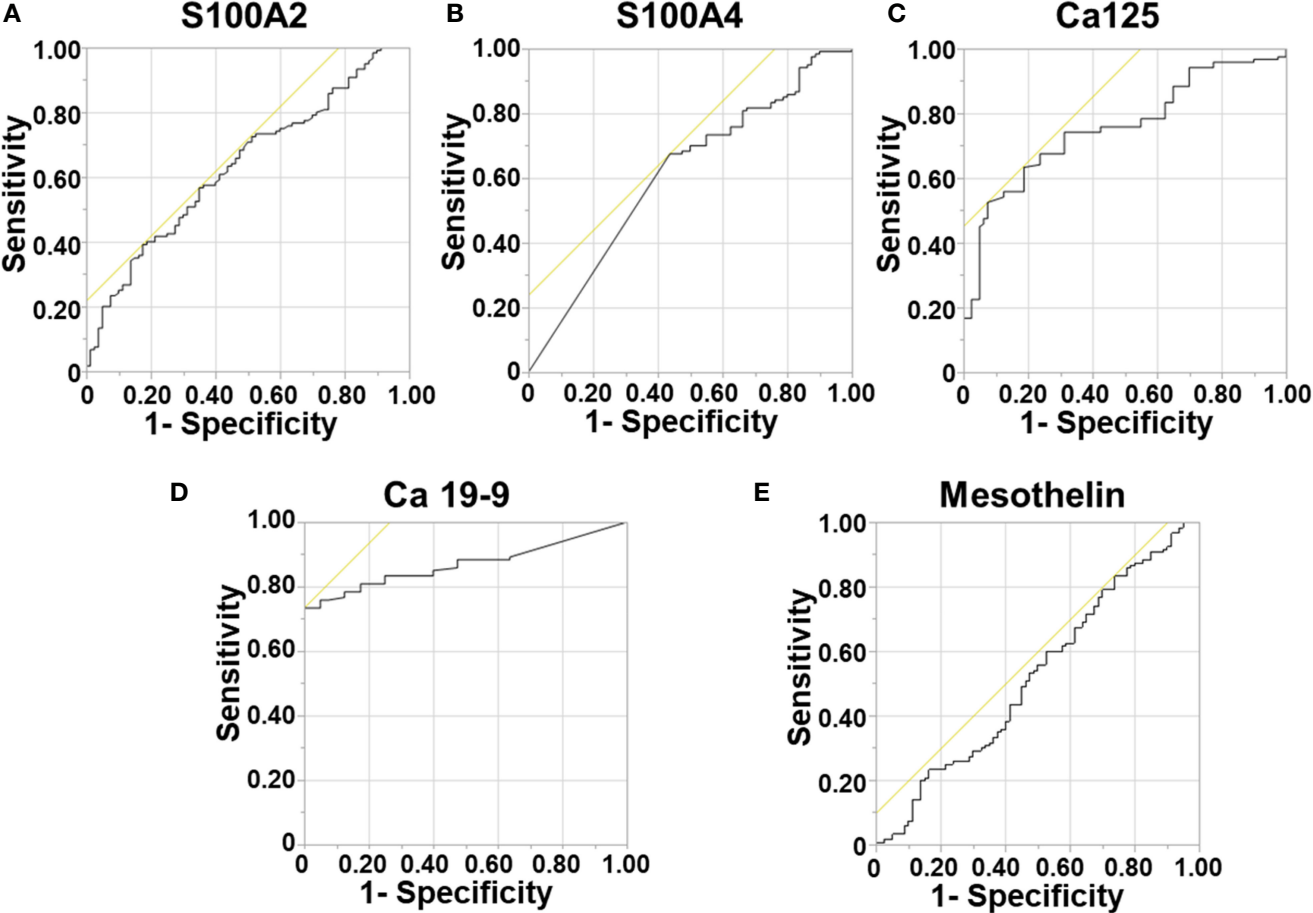
Diagnostic Ability of Biomarkers. Receiver Operator Curves were generated to determine the diagnostic potential of individual biomarkers. for: **(A)** S100A2; **(B)** S100A4; **(C)** Ca-125; **(D)** Ca 19-9; and **(E)** Mesothelin.

Initially, the ability of each biomarker (*i.e.*, S100A4, S100A2, Mesothelin, Ca-125 and Ca 19-9) to diagnose PDAC was assessed by comparing expressions with healthy controls. Serum levels of S100A4, S100A2, Ca-125 and Ca19-9 demonstrated moderate to high ability to diagnose PDAC with AUROC values of 0.613, 0.634, 0.755 and 0.869, respectively (**Figure 1**). In contrast, mesothelin showed poor diagnostic ability (AUROC: 0.525; **Figure 1**).

Next, a multivariable model for a panel of S100A4, S100A2, Ca-125 and Ca 19-9 was generated to assess its diagnostic ability. A diagnostic cut-off based on optimum sensitivity and selectivity was selected for diagnostic multivariable model. The cut-off, sensitivity and selectivity values are described in **Supplementary Table 1**. The panel showed very high diagnostic ability (AUROC: 0.913; **Supplementary Table 3**), which was superior to the current clinically used biomarker Ca 19-9 alone (AUROC: 0.869).

### Survival Analysis Based on Serum Biomarker Levels

**Figure 2:**
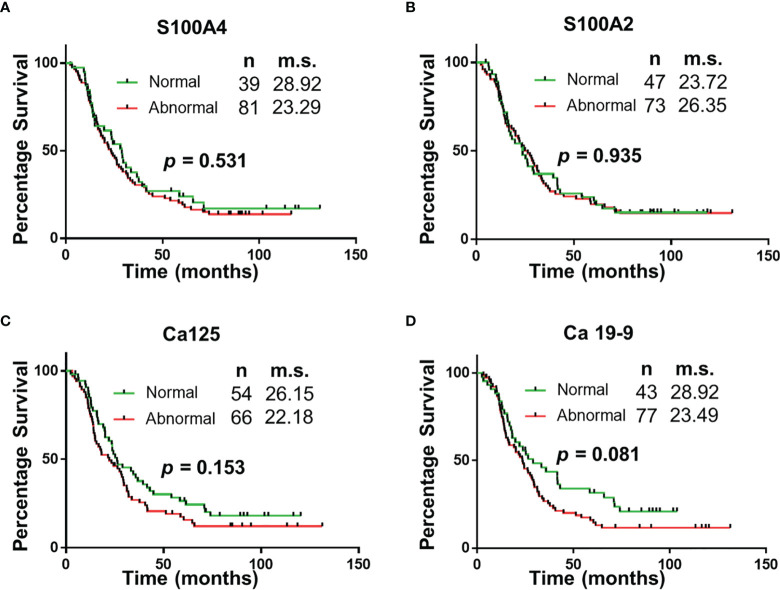
Univariable Survival Analysis of Individual Biomarkers. **(A–D)** Kaplan Meier survival curves for individual biomarkers were generated using prognostic cut-offs (**Supplementary Table 3**). n, number of patients; m.s., median survival in months.

Survival correlation with abnormal serum biomarker levels were determined using Kaplan Meier curves. Abnormal serum levels of S100A4 (median survival (m.s.): 28.92 vs 23.29 months; **Figure 2**), Ca-125 (m.s.: 26.15 vs 22.18 months; **Figure 2**) and Ca19-9 (m.s.: 28.92 vs 23.49 months; **Figure 2**) led to reduction in the median overall survival time. In contrast, abnormal serum levels of S100A2 resulted in increased median survival time (m.s.: 23.72 vs 26.35 months; **Figure 2**). However, none of the biomarkers individually corresponded with overall survival.

**Figure 3:**
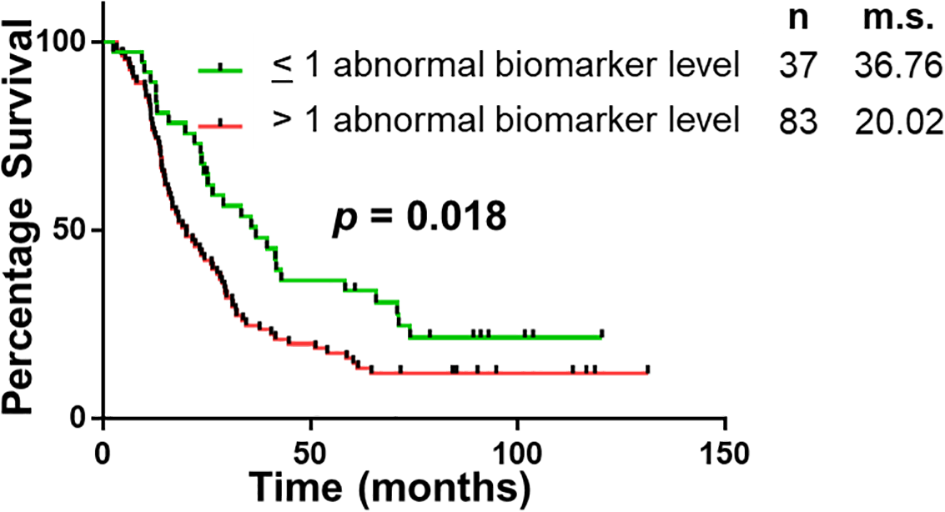
Univariable Survival Analysis of Biomarker Panel. Kaplan Meier survival curves comparing patients with abnormal biomarker levels of one or less biomarker and patients with abnormal biomarker levels of two or more biomarkers. n, number of patients; m.s., median survival in months.

The panel of S100A4, Ca-125 and Ca 19-9 was further analysed to determine its ability to stratify patients based on their overall survival. Initially, patients were divided into four groups: (1) none of the biomarkers with abnormal levels (n = 6); (2) one biomarker with abnormal levels (n = 31); (3) two biomarkers with abnormal levels (n = 56); (4) three biomarkers with abnormal levels (n = 27). Multiple comparison Kaplan Meier curve analysis did not achieve statistical significance (p = 0.121; **Supplementary Figure 1**), potentially due to very small number of patients in some categories. The combination of first two and last two categories was able to stratify patients based on their overall survival (**Figure 3**). The patients with abnormal levels of one or less of the biomarker (n = 37) had significantly improved survival outcomes, compared to those with abnormal levels of two or more biomarkers (n = 83; m.s.: 36.76 vs 20.02 months, p = 0.018; **Figure 3**). Patient distribution based on tumour characteristics was also analysed (**Supplementary Table 4**, which showed uniform distribution in both biomarker groups.

## Discussion

The study demonstrates that of the select group of biomarkers included in this study, a panel of four (S100A4, S100A2, Ca-125 and Ca 19-9) have superior diagnostic potential compared to the current biomarker used in clinical practice, Ca 19-9 alone. Additionally, the abnormal expression of two or more biomarkers correlated with worse survival (median survival: 36.76 vs 20.02 months; p < 0.05). The utility of this biomarker panel in the accurate diagnosis of PDAC and implications of biomarker expression on prognosis may assist with personalization of treatment and improved survival outcomes.

PDAC has one of the lowest rates of survival with a 5-year survival of between 5-10% (7–9). Most patients are diagnosed with an advanced disease stage, and of the 15-20% of patients who are candidates for surgical resection with curative intent at the time of diagnosis (10), more than 50% recur within 12 months of surgery (11). Survival rates in PDAC have changed little over the last 50 years (8), highlighting the complexity of accurate diagnosis and limitations of treatment. This failure of treatment highlights the inability of current decision-making strategies, which are primarily radiological and clinical, to accurately stratify patients into different prognostic groups based on actual tumour biology. Part of the reason is that it is often difficult to obtain adequate amount of tumour tissue sample for analysis and the associated costs of genetic analysis. Identification of a prognostic biomarker signature in pre-operative PDAC patient’s blood will help clinicians recommend informed decisions regarding the appropriate treatment strategy for each individual patient and has potential to markedly improve the standard of care for these patients. The ability to select patients for personalized neoadjuvant chemotherapy will revolutionize care for PDAC. For example, it was recently reported that combinations of SRC proto-oncogene or mitogen-activated protein kinase 1/2 inhibitors with gemcitabine possess synergistic effects on the squamous subtype of PDAC cells which correlates with a triple positive on our tissue biomarker panel (12).

The identification of specific serum biomarkers that have diagnostic and prognostic potential is of high utility in accurate diagnosis and improving survival outcomes (13). Biomarker expression in serum is easily obtainable in the form of a liquid biopsy of the patient’s blood. This test eradicates factors such as cost, availability of expertise, equivocal results as seen in cases of inadequate tissue sampling from FNA or ductal brushings, risk of injury to intra-abdominal structures, acute pancreatitis and potential for needle-track seeding (14). Our study reveals a panel of biomarkers that can be utilized as a method of accurate diagnosis of PDAC. Ca 19-9 is a biomarker that is most widely used in diagnosis and monitoring progression of PDAC. Our novel biomarker panel has a sensitivity and specificity which has been demonstrated to be superior to the presently used tumor marker Ca 19-9. However, this increase was only modest (AUROC: 0.913 *vs* 0.869) and a future larger multi-institutional study will be required to further corroborate these findings.

Looking ahead, there are other potential applications for a liquid biopsy panel for PDAC. For example, there are a certain percentage of patients with intraductal papillary mucinous neoplasm (IPMN) and mucinous cystic neoplasms (MCN) that have an associated ductal adenocarcinoma. It is often difficult to accurately select patients for surgery *vs* observation, and this group may be suitable for liquid serum biopsy in attempt to gain an accurate diagnosis of PDAC to improve survival outcomes. The biomarker panel may also have utility in the follow-up of resected PDAC patients to diagnose recurrence early. These applications will be the focus of future studies.

The major limitation of this study is the retrospective nature of this analysis, a relatively smaller cohort, lack of external validation cohort, only a single timepoint for analysis at the time of surgery and lack of other comparator groups for determining diagnostic accuracy (*e.g.*, patients with pancreatitis). Future, multi-institutional cohort with prospective design would serve to further validate this identified biomarker panel for PDAC prognosis. In addition, future studies will also involve serum specimens collected at multiple longitudinal time points and will include patients with other benign pancreatic conditions such as pancreatitis.

In conclusion, this study forms a critical basis for the future development of a minimally invasive blood test for accurate diagnosis and prognostication of PDAC patients.

## Data Availability Statement

The raw data supporting the conclusions of this article will be made available by the authors, without undue reservation.

## Ethics Statement

The studies involving human participants were reviewed and approved by Northern Sydney Local Health District HREC. The patients/participants provided their written informed consent to participate in this study.

## Author Contributions

SM, NB, AM, and SS were involved in analysis and interpretation of data. SS, JS, and AM contributed to conception of idea and design. All authors contributed to the article and approved the submitted version.

## Funding

This project was supported by Philanthropic funding received by AM and JS from the RT Hall Trust.

## Conflict of Interest

The authors declare that the research was conducted in the absence of any commercial or financial relationships that could be construed as a potential conflict of interest.

## References

[1] Rahib L Smith BD Aizenberg R Rosenzweig AB Fleshman JM Matrisian LM. Projecting Cancer Incidence and Deaths to 2030: The Unexpected Burden of Thyroid, Liver, and Pancreas Cancers in the United States. Cancer Res (2014) 74:2913–21. doi: 10.1158/0008-5472.CAN-14-0155 24840647

[2] Sung H Ferlay J Siegel RL Laversanne M Soerjomataram I Jemal A. Global Cancer Statistics 2020: GLOBOCAN Estimates of Incidence and Mortality Worldwide for 36 Cancers in 185 Countries. CA Cancer J Clin (2021) 71:209–249. doi: 10.3322/caac.21660 33538338

[3] Bailey P Chang DK Nones K. Genomic Analyses Identify Molecular Subtypes of Pancreatic Cancer. Nature (2016) 531:47–52. 10.1038/nature16965 26909576

[4] Hoyer K Hablesreiter R Inoue Y Yoshida K Briest F Christen F. A Genetically Defined Signature of Responsiveness to Erlotinib in Early-Stage Pancreatic Cancer Patients: Results From the CONKO-005 Trial. EBioMedicine (2021) 66:103327. doi: 10.1016/j.ebiom.2021.103327 33862582 PMC8054140

[5] Nahm CB Turchini J Jamieson N Moon E Sioson L Itchins M. Biomarker Panel Predicts Survival After Resection in Pancreatic Ductal Adenocarcinoma: A Multi-Institutional Cohort Study. Eur J Surg Oncol (2019) 45:218–24. doi: 10.1016/j.ejso.2018.10.050 30348604

[6] Sahni S Moon EA Howell VM Mehta S Pavlakis N Chan D. Tissue Biomarker Panel as a Surrogate Marker for Squamous Subtype of Pancreatic Cancer. Eur J Surg Oncol (2020) 46:1539–42. doi: 10.1016/j.ejso.2020.02.001 32061458

[7] Dreyer SB Chang DK Bailey P Biankin AV. Pancreatic Cancer Genomes: Implications for Clinical Management and Therapeutic Development. Clin Cancer Res (2017) 23:1638–46. doi: 10.1158/1078-0432.CCR-16-2411 28373362

[8] Ansari D Torén W Zhou Q Hu D Andersson R. Proteomic and Genomic Profiling of Pancreatic Cancer. Cell Biol Toxicol (2019) 35:333–43. doi: 10.1007/s10565-019-09465-9 PMC6757097 30771135

[9] Zhou B Xu J-W Cheng Y-G Gao J-Y Hu S-Y Wang L. Early Detection of Pancreatic Cancer: Where Are We Now and Where Are We Going? Int J Cancer (2017) 141:231–41. doi: 10.1002/ijc.30670 28240774

[10] Klaiber U Hackert T. Conversion Surgery for Pancreatic Cancer—The Impact of Neoadjuvant Treatment. Front Oncol (2020) 9:1501. doi: 10.3389/fonc.2019.01501 PMC6971165 31993372

[11] Groot VP Rezaee N Wu W Cameron JL Fishman EK Hruban RH. Patterns, Timing, and Predictors of Recurrence Following Pancreatectomy for Pancreatic Ductal Adenocarcinoma. Ann Surg (2018) 267:936–45. doi: 10.1097/SLA.0000000000002234 28338509

[12] Er JL Goh PN Lee CY Tan YJ Hii L-W Mai CW. Identification of Inhibitors Synergizing Gemcitabine Sensitivity in the Squamous Subtype of Pancreatic Ductal Adenocarcinoma (PDAC). Apoptosis (2018) 23:343–55. doi: 10.1007/s10495-018-1459-6 29740790

[13] Ideno N Mori Y Nakamura M Ohtsuka T. Early Detection of Pancreatic Cancer: Role of Biomarkers in Pancreatic Fluid Samples. Diagnostics (2020) 10:1056. doi: 10.3390/diagnostics10121056 PMC7762187 33291257

[14] Tsutsumi H Hara K Mizuno N Hijioka S Imaoka H Tajika M. Clinical Impact of Preoperative Endoscopic Ultrasound-Guided Fine-Needle Aspiration for Pancreatic Ductal Adenocarcinoma. Endoscopic Ultrasound (2016) 5:94–100. doi: 10.4103/2303-9027.180472 27080607 PMC4850801

