# Corrigendum: Serum Biomarker Panel for Diagnosis and Prognosis of Pancreatic Ductal Adenocarcinomas

**DOI:** 10.3389/fonc.2021.774861

**Published:** 2021-10-06

**Authors:** Shreya Mehta, Nazim Bhimani, Anthony J. Gill, Jaswinder S. Samra, Sumit Sahni, Anubhav Mittal

**Affiliations:** ^1^ Northern Clinical School, Faculty of Medicine and Health, University of Sydney, Sydney, NSW, Australia; ^2^ Kolling Institute of Medical Research, University of Sydney, Sydney, NSW, Australia; ^3^ Upper Gastro Intestinal (GI) Surgical Unit, Royal North Shore Hospital and North Shore Private Hospital, Sydney, NSW, Australia; ^4^ Cancer Diagnosis and Pathology Group, Kolling Institute of Medical Research, Royal North Shore Hospital, St Leonards, NSW, Australia; ^5^ Australian Pancreatic Centre, Sydney, NSW, Australia; ^6^ NSW Health Pathology, Department of Anatomical Pathology, Royal North Shore Hospital, St Leonards, NSW, Australia

**Keywords:** pancreatic ductal adenocarcinoma, diagnostic biomarkers, prognostic biomarkers, S100A4, Ca-125 and Ca 19-9, survival analysis

In the original article, there was a mistake in **Figures 2**, **3**, **Supplementary Figure 1** and **Table 1**. There was an error in the survival data for some patients which has slightly modified curves. The corrected **Figures 2**, **3**, **Supplementary Figure 1** and **Table 1** appears below.

**Figure 2:**
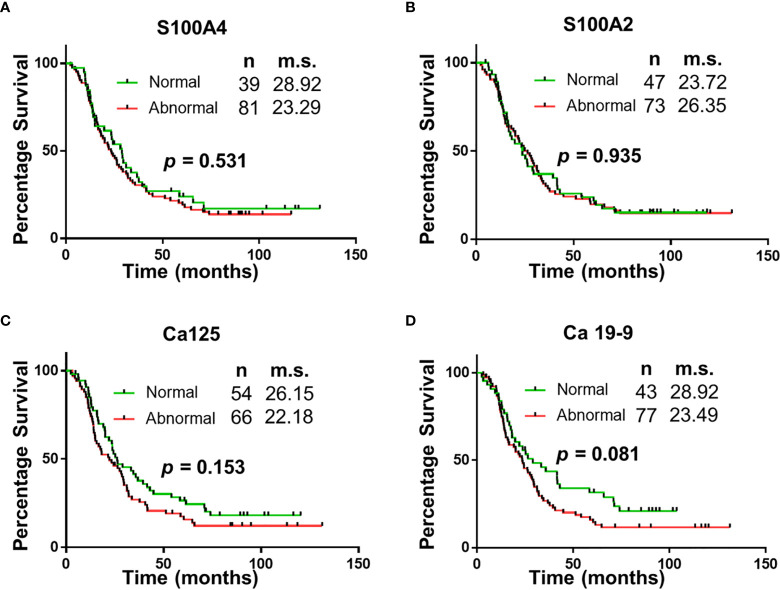
Univariable Survival Analysis of Individual Biomarkers. **(A–D)** Kaplan Meier survival curves for individual biomarkers were generated using prognostic cut-offs (**Supplementary Table 3**). n, number of patients; m.s., median survival in months.

**Figure 3:**
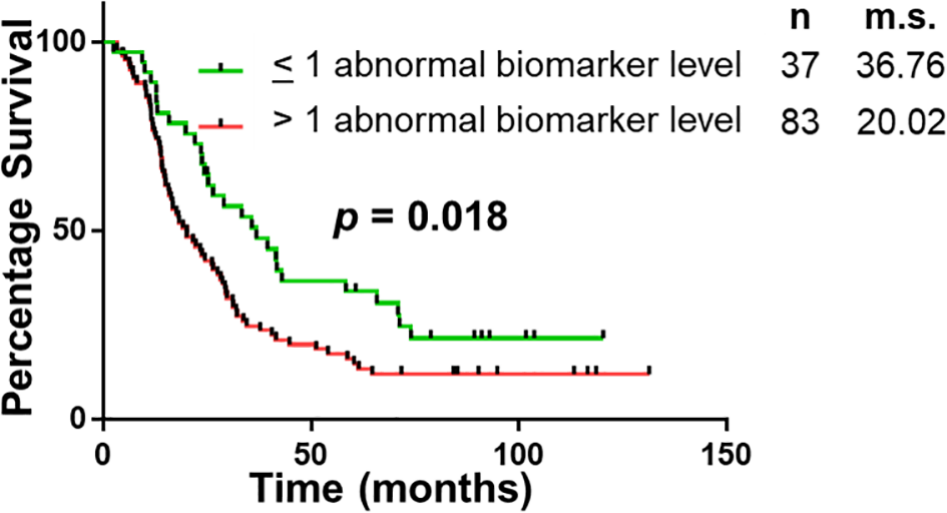
Univariable Survival Analysis of Biomarker Panel. Kaplan Meier survival curves comparing patients with abnormal biomarker levels of one or less biomarker and patients with abnormal biomarker levels of two or more biomarkers. n, number of patients; m.s., median survival in months.

**Table 1:** Patient and tumour characteristics and correlation with survival status.

	**Total n (%)**	**HR**	**95% CI**	***p*-value**
Age				0.237
<70 years	72 (60.0)	Reference		
≥70 years	48 (40.0)	1.27	0.85-1.90	
Gender				0.706
Male	61 (50.8)	Reference		
Female	59 (49.2)	0.93	0.62-1.38	
Tumour size				0.009
<35mm	55 (45.8)	Reference		
≥35mm	65 (54.2)	1.70	1.14-2.55	
T Stage				0.041
T1 & T2	9 (7.5)	Reference		
T3 & T4	111 (92.5)	2.56	1.04-6.33	
Node Positive				0.001
No	26 (21.7)	Reference		
Yes	94 (78.3)	2.68	1.51-4.76	
Vascular Invasion				<0.001
No	46 (38.3)	Reference		
Yes	74 (61.7)	2.49	1.61-3.87	
Perineural Invasion				0.024
No	38 (31.7)	Reference		
Yes	82 (68.3)	1.66	1.07-2.58	
Grade				0.021
0 or 1	84 (70.0)	Reference		
2 or 3	36 (30.0)	1.65	1.08-2.54	
Blood loss				0.873
<450mL	52 (43.3)	Reference		
≥450mL	68 (56.7)	1.03	0.69-1.54	
Length of stay				0.347
<12 days	45 (37.5)	Reference		
≥12 days	75 (62.5)	0.82	0.54-1.24	
Margin Status				0.002
R0	49 (40.8)	Reference		
R1	71 (59.2)	1.89	1.25-2.85	

In the original article, there was an error. The stated median survival and p value of survival analysis were incorrect. A correction has been made to ***Results, Survival Analysis Based on Serum Biomarker Levels paragraph 1 and 2:***

“Survival correlation with abnormal serum biomarker levels were determined using Kaplan Meier curves. Abnormal serum levels of S100A4 (median survival (m.s.): 28.92 *vs* 23.29 months; **Figure 2**), Ca-125 (m.s.: 26.15 *vs* 22.18 months; **Figure 2**) and Ca19-9 (m.s.: 28.92 *vs* 23.49 months; **Figure 2**) led to reduction in the median overall survival time. In contrast, abnormal serum levels of S100A2 resulted in increased median survival time (m.s.: 23.72 *vs* 26.35 months; **Figure 2**). However, none of the biomarkers individually corresponded with overall survival.

The panel of S100A4, Ca-125 and Ca 19-9 was further analysed to determine its ability to stratify patients based on their overall survival. Initially, patients were divided into four groups: (1) none of the biomarkers with abnormal levels (n = 6); (2) one biomarker with abnormal levels (n = 31); (3) two biomarkers with abnormal levels (n = 56); (4) three biomarkers with abnormal levels (n = 27). Multiple comparison Kaplan Meier curve analysis did not achieve statistical significance (p = 0.121; **Supplementary Figure 1**), potentially due to very small number of patients in some categories. The combination of first two and last two categories was able to stratify patients based on their overall survival (**Figure 3**). The patients with abnormal levels of one or less of the biomarker (n = 37) had significantly improved survival outcomes, compared to those with abnormal levels of two or more biomarkers (n = 83; m.s.: 36.76 *vs* 20.02 months, p = 0.018; **Figure 3**). Patient distribution based on tumour characteristics was also analysed (**Supplementary Table 4**), which showed uniform distribution in both biomarker groups”.

In the original article, there was an error. The stated median survival was incorrect. A correction has been made to ***Discussion, paragraph 1***:

“The study demonstrates that of the select group of biomarkers included in this study, a panel of four (S100A4, S100A2, Ca-125 and Ca 19-9) have superior diagnostic potential compared to the current biomarker used in clinical practice, Ca 19-9 alone. Additionally, the abnormal expression of two or more biomarkers correlated with worse survival (median survival: 36.76 *vs* 20.02 months; p < 0.05). The utility of this biomarker panel in the accurate diagnosis of PDAC and implications of biomarker expression on prognosis may assist with personalization of treatment and improved survival outcomes”.

The authors apologize for these errors and state that this does not change the scientific conclusions of the article in any way. The original article has been updated.

## Publisher’s Note

All claims expressed in this article are solely those of the authors and do not necessarily represent those of their affiliated organizations, or those of the publisher, the editors and the reviewers. Any product that may be evaluated in this article, or claim that may be made by its manufacturer, is not guaranteed or endorsed by the publisher.

