# Supplementary material for: Serum Biomarker Panel for Diagnosis and Prognosis of Pancreatic Ductal Adenocarcinomas
Source: Front Oncol. 2021 Jul 5;11:708963. doi: 10.3389/fonc.2021.708963 (PMC8287202; doi:10.3389/fonc.2021.708963)
Supplement: Supplementary file 1 [file DataSheet_1.docx]

**SUPPLEMENTARY** **DATA**

**Supplementary Table 1:** Diagnostic Cut-off values

| **Biomarker** | **Cut-off value** | **Sensitivity at Cut-off** | **Specificity at Cut-off** |
| --- | --- | --- | --- |
| **S100A2** | >2.85 | 72.5% | 48.75% |
| **Ca125** | >12.1 | 74.17% | 68.75% |
| **Ca19-9** | >9.1 | 80.83% | 82.5% |
| **S100A4** | <0.0059 | 67.5% | 55% |

**Supplementary Table 2:** Prognostic Cut-off values

| **Biomarker** | **Cut-off value** | **Sensitivity at Cut-off** | **Specificity at Cut-off** |
| --- | --- | --- | --- |
| **S100A2** | >3.3783 | 60.83% | 58.875% |
| **Ca125** | >16.1 | 55.83% | 87.5% |
| **Ca19-9** | >37 | ~65% | 100% |
| **S100A4** | <0.0059 | 67.5% | 55% |

**Supplementary Table 3:** Multivariable Diagnostic Model for four selected biomarkers, *i.e.,* S100A2, S100A4, Ca125 and Ca 19-9.


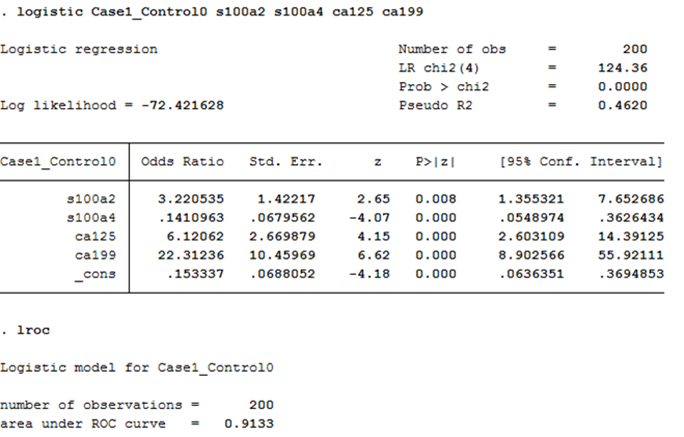


**Supplementary Table 4:** Distribution of Patient in Prognostic Biomarker Groups (0-1 abnormal vs 2-3 abnormal) based on Tumour Characteristics.

|  | **<1 Abnormal Biomarker Level** | **>1 Abnormal Biomarker Level** |
| --- | --- | --- |
| Tumour size  <35mm  ≥35mm | 20 (54.1%)  17 (45.9%) | 35 (42.2%)  48 (57.8%) |
| T Stage  T1 & T2  T3 & T4 | 03 (8.1%)  34 (91.9%) | 06 (7.2%)  77 (92.8%) |
| Node Positive  No  Yes | 09 (24.3%)  28 (75.7%) | 17 (20.5%)  66 (79.5%) |
| Vascular Invasion  No  Yes | 16 (43.2%)  21 (56.8%) | 30 (36.1%)  53 (63.9%) |
| Perineural Invasion  No  Yes | 13 (35.1%)  24 (64.9%) | 25 (30.1%)  58 (69.9%) |
| Grade  0 or 1  2 or 3 | 01 (2.7%)  36 (97.3%) | 03 (3.6%)  80 (96.4%) |
| Blood loss  <450mL  ≥450mL | 18 (48.6%)  19 (51.4%) | 34 (41%)  49 (59%) |
| Length of stay  <12 days  ≥12 days | 15 (40.5%)  22 (59.5%) | 30 (36.1%)  53 (63.9%) |
| Margin Status  R0  R1 | 16 (43.2%)  21 (56.8%) | 33 (39.8%)  50 (60.2%) |

**Supplementary Figure 1: Survival Analysis of Biomarker Panel**. Multiple Comparison Kaplan Meier survival curves comparing patients with abnormal levels of none, one, two or all three biomarkers. n = number of patients; m.s. = median survival in months.

**
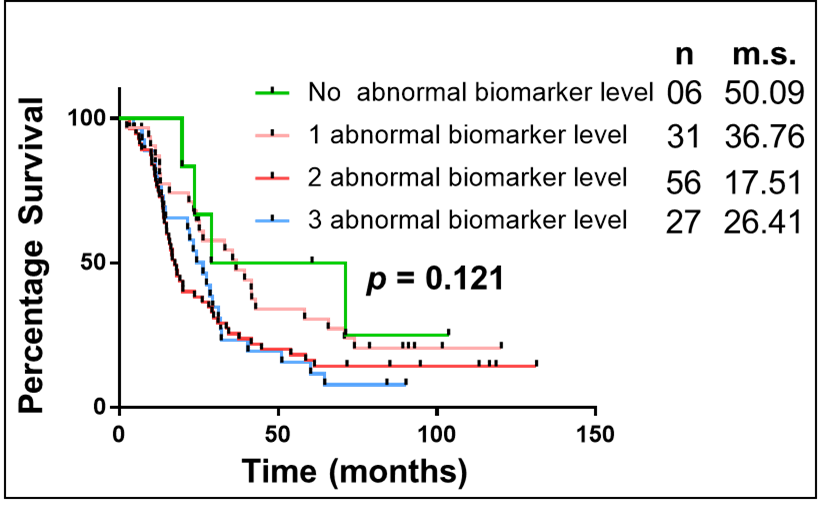
**
